# Trajectory of medical expenditure and regional disparities in hypertensive patients in South Korea

**DOI:** 10.3389/fpubh.2024.1294045

**Published:** 2024-06-13

**Authors:** Gi-Hyun Kim, Ji-Soo Song, Ji-Woong Nam, Woo-Ri Lee, Ki-Bong Yoo

**Affiliations:** ^1^Institute of Health and Welfare, Yonsei University, Wonju, Republic of Korea; ^2^Department of Health Administration, Yonsei University Graduate School, Wonju, Republic of Korea; ^3^Department of Research and Analysis, National Health Insurance Service Ilsan Hospital, Goyang-si, Republic of Korea; ^4^Division of Health Administration, College of Software and Digital Healthcare Convergence, Yonsei University, Wonju, Republic of Korea

**Keywords:** hypertension, regional disparities, medical expenditure, trajectory, inverse probability weighting (IPW)

## Abstract

The aim of this study is to understand how different regions influence the management and financial burden of hypertension, and to identify regional disparities in hypertension management and medical expenditure. The study utilized data from the Korean Health Panel Survey conducted between 2014 and 2018, focusing on individuals with hypertension. Medical expenditures were classified into three trajectory groups: “Persistent Low”, “Expenditure Increasing”, and “Persistent High” over a five-year period using trajectory analysis. Inverse Probability Weighting (IPW) analysis was then employed to identify the association between regions and medical expenditure trajectories. The results indicate that individuals residing in metropolitan cities (Busan, Daegu, Incheon, Gwangju, Daejeon, and Ulsan) (OR = 1.07; 95% CI: 1.03-1.12) and rural areas (OR = 1.07; 95% CI: 1.03-1.11) were more likely to belong to the “Expenditure Increasing” group compared to the “Persistent Low Expenditure” group, as opposed to those in the capital city (Seoul). Additionally, residents of rural areas (OR = 1.05; 95% CI: 1.01-1.08) were more likely to be in the “High Expenditure” group compared to the “Persistent Low Expenditure” group than those residing in the capital city. These findings suggest that individuals in rural areas may be receiving relatively inadequate management for hypertension, leading to higher medical expenditures compared to those in the capital region. These disparities signify health inequality and highlight the need for policy efforts to address regional imbalances in social structures and healthcare resource distribution to ensure equitable chronic disease management across different regions.

## 1 Introduction

Since 2021, chronic diseases have accounted for 79.6% of all deaths in Korea. Specifically, cardiovascular diseases resulted in 54,176 deaths, representing 17.0% of the total. Deaths due to diabetes, chronic obstructive pulmonary disease (COPD), and malignant neoplasms (cancer) accounted for 8,961 (2.8%), 14,005 (4.4%), and 82,688 (26.0%) of deaths, respectively (1). Since 2020, chronic disease-related medical expenditures in Korea have amounted to 71 trillion KRW, making up 85.0% of the total medical expenditures. Furthermore, these expenditures increased from 10.4 trillion KRW in 2009 to 22.9 trillion KRW in 2020, indicating a significant rise of 12.5 trillion KRW and demonstrating a continuous upward trend (1).

Effective management of chronic diseases at the primary care level can reduce unnecessary hospitalizations, particularly for conditions such as hypertension and diabetes (2). Korea’s hospitalization rate due to chronic diseases is notably high compared to other Organization for Economic Cooperation and Development (OECD) countries. The hospitalization rate for hypertension stands at 129.8 per 100,000 population, significantly higher than the OECD average of 74.3 per 100,000 (3). These high hospitalization rates can be attributed to inadequate management of chronic illnesses at the primary care level and a hospital system predominantly centered on the private sector, leading to relatively easy hospital admissions. Consequently, the OECD has recommended the establishment of a robust community-based primary care system in South Korea, alongside national support, investment, quality assessment, and value-based incentives for primary care (4).

Health insurance in South Korea is broadly classified into National Health Insurance (NHI) and complementary private health insurance. The basic framework of Korea’s health insurance system is the NHI program, which was established by the Medical Insurance Act in 1963 to provide a social safety net for all citizens. By 1989, virtually all citizens were required to be covered by the NHI program. Complementary private health insurance, on the other hand, is a financial product available for purchase by individuals who have the willingness and means to buy it in the financial market. This private insurance serves as a payment method for medical expenses partially covered by NHI and helps to reduce the financial burden on individuals and their families (5).

Researchers have consistently highlighted the excessive use of medical services by subscribers of complementary private health insurance. Studies on insurance subscription and moral hazard among these subscribers generally conclude that moral hazard arises because insured individuals, having transferred the risk, do not bear it themselves unless appropriate control measures are implemented. In particular, adverse selection and moral hazard are prevalent among private health insurance subscribers. In South Korea, there is also evidence of moral hazard and excessive use of medical services among these subscribers. This excessive use is partly due to a low awareness of the costs associated with medical services, but it is also significantly driven by incentives from some providers to encourage higher consumption of medical services (5).

The provision of medical services in Korea reveals that public medical institutions have an average nationwide share of 11.0%, while private medical institutions dominate with an 89% share. Compared to major OECD countries, Korea’s total number of medical institutions stands at 3,924, surpassing the OECD average of 1,253. However, whereas 51.79% of medical institutions in OECD countries are public, only 5.71% of Korean medical institutions fall into this category. Conversely, private medical institutions make up 94.29% of the total in Korea, compared to the OECD average of 44.48%, which includes 16.38% non-profit and 28.10% for-profit institutions (6).

Although categorized as non-profit, Korean private medical institutions tend to promote medical consumption to ensure profitability. Additionally, there have been ongoing concerns about the declining quality of care in public medical institutions. As a result, patients seeking higher-quality medical services often prefer private medical institutions (6).

Primary care patients in Korea tend to choose medical facilities according to their preferences, supported by the National Health Insurance (NHI) system. The characteristics of Korea’s insurance system and the structure of medical facilities interact, making hospitalization in the private sector relatively accessible (7).

Hypertension can lead to various cardiovascular diseases, including coronary artery disease, heart failure, stroke, and vascular dementia. In 2021, cardiovascular diseases, including hypertension, were the second leading cause of death in South Korea, with a mortality rate of 121.5 per 100,000 population (1). The number of hypertension patients aged 20 and above in South Korea increased significantly, from 7.08 million in 2007 to 13.74 million in 2021. Since 2018, the proportion of male hypertension patients has slightly surpassed that of females, with males and females accounting for 51.1 and 48.9%, respectively, in 2021. The age-standardized prevalence rate, adjusted for the aging population, rose from 22.9% in 2007 to 27.7% in 2021, underscoring hypertension as a critical disease requiring national management (8). Furthermore, hypertension is particularly dangerous when it coexists with other conditions. Previous studies have demonstrated that the number of chronic diseases individuals have influences their healthcare utilization (9).

Effective management of hypertension requires continuous care and timely intervention at the primary care level. Since 2005, South Korea has implemented several national-level projects for managing chronic diseases, including cardiovascular diseases (10). Initiated with the 2007 pilot program for high-risk cardiovascular registration and management in Daegu Metropolitan City by the Korea Disease Control and Prevention Agency, this program has expanded to over 30 public health centers nationwide. In 2014, a community-based primary care pilot program was introduced and implemented through health insurance payments. In 2016, the chronic disease management payment pilot project was launched to manage patients with hypertension and diabetes, involving 1,870 clinics nationwide. Since 2019, the primary care-centered chronic disease management pilot project has been ongoing, complementing existing systems (3, 11). These efforts have helped control the increase in chronic diseases and reduce the occurrence of complications that worsen conditions (12).

Local conditions significantly influence the self-management of hypertension, and personal or societal health disparities can arise due to regional differences in access to healthcare resources. Recent studies have highlighted the exacerbation of population decline issues in certain regions, indicating the need for customized regional gap-reduction projects to ensure equity in chronic disease management across different areas (13). Identifying regional disparities in chronic diseases and understanding local contexts are essential for systematically performing chronic disease management (14).

While South Korea’s overall population health has improved, studies on regional disparities in health, including disease prevalence, mortality rates, and health behaviors between urban and rural areas, as well as between metropolitan and non-metropolitan areas, have consistently shown an increase (15). Jun and Kang (15) reported that spatial inequalities arise due to regional characteristics. Additionally, Han and Kim (16) demonstrated that cardiovascular disease risk and healthcare utilization varied significantly between low and high population-density regions, suggesting that regional characteristics substantially impact healthcare outcomes and expenditures. These findings underscore the importance of understanding regional variations to develop effective public health strategies and policies.

Although previous studies have aimed to identify specific regional differences, research specifically addressing the long-term trends of region-centered chronic diseases remains limited. Therefore, this study aimed to explore the relationship between regions and medical expenditure trajectories for patients with hypertension, considering the perspective of regional disparities in medical expenditure over time.

This study focuses on investigating whether correlations exist between regions and the medical expenditures incurred by patients with hypertension over time. By examining the patterns of hypertension-related medical expenditures across regions, this study aims to gain insights into how regional factors may influence the management and financial burden of hypertension in South Korea. The findings of this research are intended to contribute to a better understanding of chronic disease management and medical expenditure-related regional disparities, which may help policymakers and healthcare professionals design targeted interventions and strategies to improve health outcomes and reduce disparities in hypertension care across regions.

## 2 Methods

### 2.1 Data and study population

This study utilized annual data from the Korean Health Panel Survey (KHPS) conducted between 2014 and 2018 (Beta version 1.7). The KHPS collects data from a nationally representative sample of households, with the initial survey in 2008 including 21,283 individuals from 7,009 households. Designed to be longitudinal, the same households are surveyed annually to track changes over time. The KHPS aims to provide fundamental information for policy development to enhance the responsiveness, accessibility, and efficiency of the national health and medical care system (17). This joint survey, conducted by the Korea Institute for Health and Social Affairs and the National Health Insurance Corporation, produces individual- and household-level statistics on various aspects, including medical expenditure and factors influencing healthcare utilization for the Korean population since 2008 (17).

**Figure 1:**
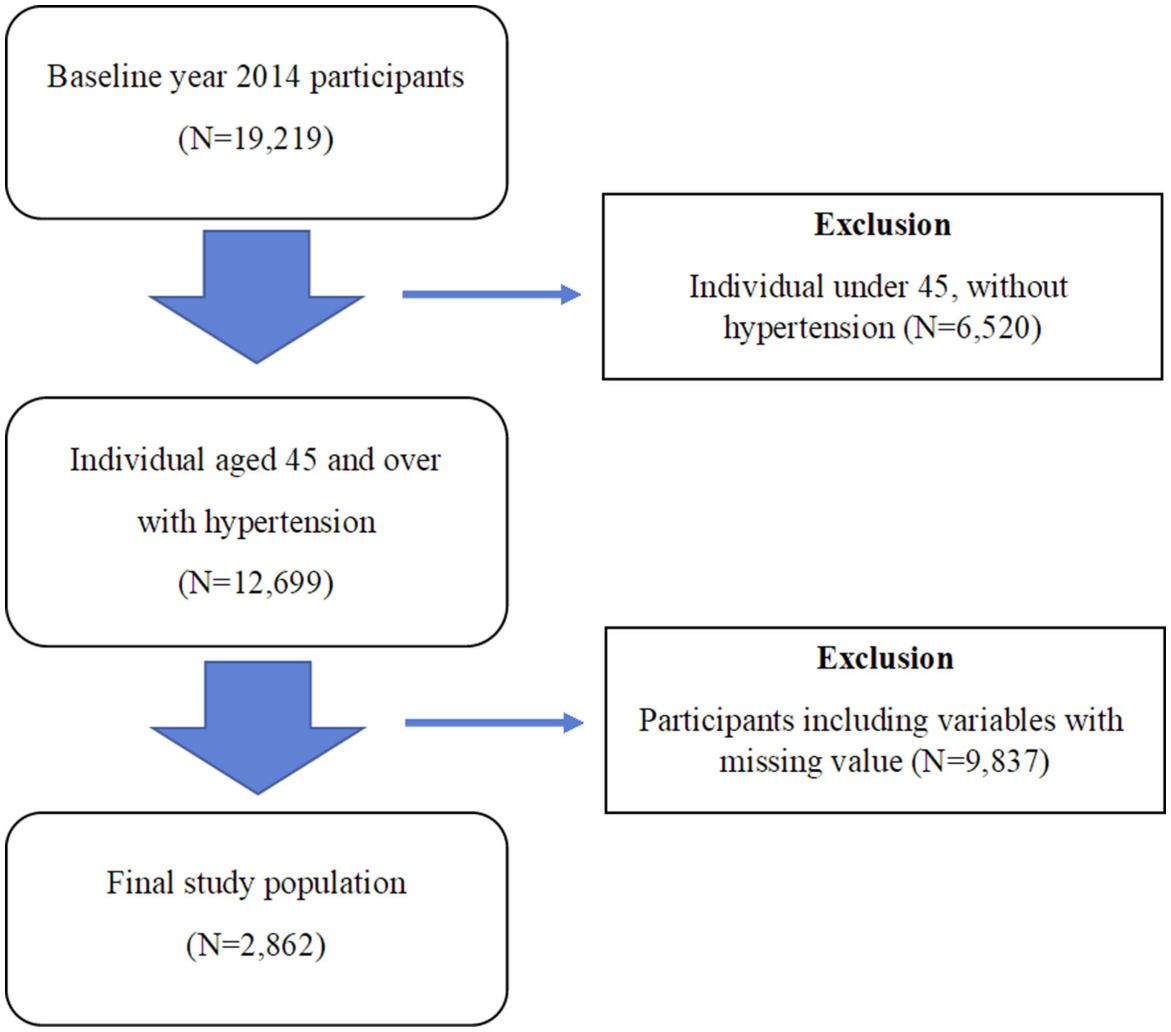
Study population flow chart.

The study followed participants over a 5-year period from 2014 to 2018. The baseline year was set as 2014, and among the 19,219 participants surveyed that year, and 6,520 participants under the age of 45 and those without hypertension were excluded. Additionally, participants with missing data for medical expenditure, type of medical insurance coverage, region and self-rated health, as well as participants with missing data for the year variable between 2014 and 2018 were excluded, resulting in the exclusion of 9,837 individuals. The final study population consisted of individuals diagnosed with hypertension in 2014, totaling 2,862 observations for the selected study participants (Figure 1).

### 2.2 Variables

In this study, the dependent variable, which is the medical expenditure trajectory of patients with hypertension, reflects factors influencing the medical utilization of patients with hypertension, including economic status and health condition. The characteristics of these variables can be examined by applying the Andersen Behavioral Model (18, 19), which categorizes them into predisposing (age and gender), enabling (economic status), and need factors (health status).

Usually, in previous studies, medical expenditures are used as the dependent variable; however, in this study, the dependent variable was the trajectory group of medical expenditures for patients with hypertension. The trajectory group of medical expenditures for patients with hypertension was categorized into three groups using trajectory analysis. It was operationally defined as follows: “Persistent Low” group was defined as “1”, “Expenditure Increasing” group as “2”, and “Persistent High” group as “3”.

The independent variable “Region” was categorized into three groups: “Capital City (Seoul)”, “Metropolitan City (Busan, Daegu, Incheon, Gwangju, Daejeon, and Ulsan)”, and “Rural Area”. The confounders included demographic factors (gender, age, and marital status), socioeconomic factors (education and income levels), and health-related factors (self-rated health status).

Gender was defined as “1” for “female”, and “2” for “male”, The age groups were categorized as follows: “45 years and older, less than 55 years”, “55 years and older, less than 65 years”, “65 years and older, less than 75 years”, and “75 years and older”. Regarding marital status, “0” was assigned for “single, widowed, separated, and divorced” and “1” for “married”. Educational level was defined as “elementary school graduate”, “middle school graduate”, and “high school graduate or above”. Income level was categorized on the basis of the quintiles of income set by the Korean Health Panel. The Income level was divided by the square root of the actual number of household members (total number of household members in the Ind file) and then categorized into quintiles from the 1st quintile (minimum) to the 5th quintile (maximum). Self-rated health status was redefined into the following three categories: “poor”, “fair”, and “good”, using the existing quintiles. Self-rated health was measured by the question, How do you perceive your health status?’. Self-rated health refers to an individual’s comprehensive evaluation of their own health in physical, psychological, physiological, and social aspects. It serves as an indicator of personal views on health conditions that are difficult to measure medically or clinically (20).

### 2.3 Statistical analysis

This study used trajectory analysis to derive a variable called the “medical expenditure group” from the trajectory analysis, which will be set as the dependent variable. The group-based trajectory modeling was used for classifying individuals into different groups on the basis of their medical expenditure trajectories over time, allowing the researchers to identify distinct patterns and trajectories within the hypertension patient population. The group-based trajectory modeling is a method that aims to identify individual characteristics of study participants and capture similar patterns that form over time for specific variables. The group-based trajectory analysis was performed using Proc Traj in SAS software (21). The fundamental assumption of the group-based trajectory modeling is that variables not affected by time are associated with the observed trajectories (patterns) through individuals belonging to each group, whereas variables that change over time are directly associated with the observed trajectories. The group-based trajectory analysis considers the effect sizes of variables not affected by time and those that change over time to calculate the probability of each individual belonging to a specific group. The number of groups is determined by performing model-fitting comparisons using the Bayesian information criterion (BIC), and the model with the smallest value is considered the best fit (21, 22).

To obtain the dependent variable, the medical expenditure trajectory groups were formed using the group-based trajectory analysis on the basis of the total medical expenditure from 2014 to 2018, with values above the 95th percentile replaced by the corresponding value at the 95th percentile, after multiplying the total medical expenditure by 1/1,000. The significance probabilities for each group from the group-based trajectory analysis were all <0.0001, indicating statistical significance. The optimal number of groups, as determined by the lowest BIC, was three, with a BIC value of −118586.7, and the participants were categorized accordingly.

After performing the group-based trajectory analysis and obtaining the medical expenditure trajectory of the grouped patients with hypertension on the basis of the initial dependent variable, a multinomial logistic regression analysis was performed to examine how the region was associated with the likelihood of belonging to a specific medical expenditure trajectory group. Subsequently, to analyze differences in medical expenditure trajectories among patients with hypertension according to their region, a doubly robust inverse probability weighting (IPW) analysis was performed, adjusting for demographic, socioeconomic, and health-related factors (Capital city, metropolitan city, and rural areas), and the average treatment effect (ATE) was calculated.

The doubly robust estimator is considered causal effects using the propensity score (PS) model for inverse probability weighting (IPW) and the outcome regression model, adjusted for covariates after applying the weights, are correctly specified (23–25).

To ensure that the weights are appropriately applied after performing IPW in the PS model, balance assessment for weights is conducted using the Standardized Mean Difference (SMD) method. SMD is the most commonly used statistic for examining the balance of covariate distributions between treatment groups (23, 26). Typically, when the absolute value of SMD is around 0.1, it is considered that the Average Treatment Effect derived from IPW analysis meets the basic conditions.

## 3 Results

**Figure 2:**
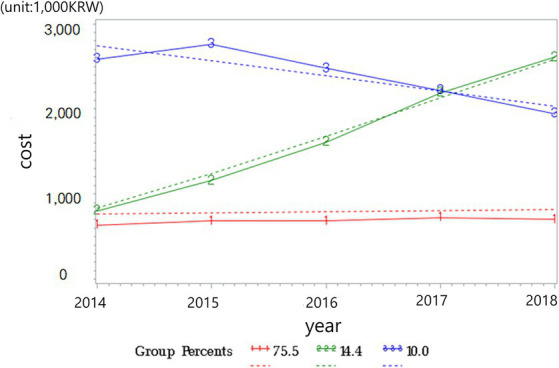
Medical expenditure trajectories.

The groups of medical expenditure trajectories identified through the group-based trajectory analysis are displayed in Figure 2.

Solid red line represent observed medical expenditure of the “Persistent Low” trajectory group; Solid green line represent observed medical expenditure of the “Expenditure Increasing” trajectory group; Solid blue line represent observed medical expenditure of the “Persistent High” trajectory group; Dotted line represent predicted medical expenditure with their 95% CI for each trajectory group.

Among the entire study population, the proportion of each group was as follows: the “Persistent Low” group comprised 76.3% (*n* = 2,185), “Expenditure Increasing” group consisted of 13.9% (*n* = 398), and “Persistent High” group accounted for 9.7% (*n* = 279). The participants in the “Persistent Low” group showed a consistent and low medical expenditure trajectory over the 5-year period from 2014 to 2018. The participants in the “Expenditure Increasing” group exhibited an increasing trend in medical expenditure over the 5-year period. The participants in the “Persistent High” group showed a consistently high medical expenditure level over the 5-year period.

The descriptive statistics results for the study participants are presented in Table 1. In the 5-year period panel data, the baseline year was set as 2014, and among the 19,219 participants surveyed in 2014. Missing values in all variables were deleted. The final study population was composed of individuals who had hypertension in panel 2014, and the total number of observations for the selected study participants was 2,862.

**Table 1:** General characteristics of the study participants.

Category	Total	Expenditure group (*n*, %)			*p*
	*n* = 2,862				
	(%)	Persistent low expenditure group	Expenditure increasing group	High expenditure group	
		(*n* = 2,185)	(*n* = 398)	(*n* = 279)	
Gender					
Male	1,217 (42.5)	970 (79.7)	146 (12.0)	101 (8.3)	0.0087
Female	1,645 (57.5)	1,215 (73.9)	252 (15.3)	178 (10.8)	
Age group (years)					
45–54	323 (11.3)	277 (85.8)	32 (9.9)	14 (4.3)	0.002
55–64	702 (24.5)	538 (76.6)	96 (13.7)	68 (9.7)	
65–74	1,108 (38.7)	818 (73.8)	167 (15.1)	123 (11.1)	
75 and up	729 (25.5)	552 (75.7)	103 (14.1)	74 (10.2)	
Education level					
Elementary school graduate	1,289 (45.0)	953 (73.9)	203 (15.7)	133 (10.4)	<0.0001
Middle school graduate	534 (18.7)	400 (74.9)	74 (13.9)	60 (11.2)	
High school graduate or above	1,039 (36.3)	832 (80.1)	121 (11.6)	86 (8.3)	
Type of medical insurance coverage					
National Health Insurance	2,632 (92.0)	1,977 (75.1)	383 (14.6)	272 (10.3)	0.0046
Medical Aid	230 (8.0)	208 (90.4)	15 (6.5)	7 (3.1)	
Marital status					
Married	2,034 (71.1)	1,539 (75.6)	278 (13.7)	217 (10.7)	0.0048
Single, separated, divorced	828 (28.9)	646 (78.0)	120 (14.5)	62 (7.5)	
Self-rated health status					
Poor	922 (32.2)	643 (69.7)	137 (14.9)	142 (15.4)	<0.0001
Fair	1,224 (42.8)	945 (77.2)	184 (15.0)	95 (7.8)	
Good	716 (25.0)	597 (83.3)	77 (10.8)	42 (5.9)	
Income level					
Low	875 (30.6)	682 (77.9)	121 (13.8)	72 (8.3)	0.0023
Under-middle	704 (24.6)	518 (73.6)	111 (15.8)	75 (10.6)	
Middle	550 (19.2)	423 (76.9)	66 (12.0)	61 (11.1)	
Upper-middle	394 (13.8)	319 (81.0)	44 (11.1)	31 (7.9)	
High	339 (11.8)	243 (71.7)	56 (16.5)	40 (11.8)	
Region^†^					
Capital city	329 (12.0)	269 (81.8)	32 (9.7)	28 (8.5)	<0.0001
Metropolitan city	783 (27.0)	598 (76.4)	113 (14.4)	72 (9.2)	
Rural area	1,750 (61.0)	1,318 (75.3)	253 (14.5)	179 (10.2)	

^†^Capital City: Seoul, Metropolitan City: Busan, Daegu, Incheon, Gwangju, Daejeon, Ulsan.

The general characteristics of the final study participants, stratified by their medical expenditure trajectory group, are shown in Table 1. The analysis showed statistically significant differences at the 95% confidence level in the characteristics of the extracted participants, including gender, age, education level, type of medical insurance coverage, marital status, subjective health perception, income level, and residential area, according to their medical expenditure trajectory group.

Of 2,862 final analyzed participants, 1,217 (42.5%) and 1,645 (57.5%) were males and females, respectively, indicating a higher proportion of females in this study. Data of patients with hypertension for 5 years up to the final survey year of 2018 showed that similar sample sizes were observed for both genders, considering only those with complete data across all surveys. Among the entire study population, the “65 to under 75 years” age group had the highest proportion at 38.7%, followed by “75 years and older” and “55 to under 65 years” with 25.5 and 24.5%, respectively. Regarding marital status, 71.1% of the participants were married. Regarding residential areas, the majority (61.0%) resided in rural areas, followed by 27.0% in the metropolitan region (Busan, Daegu, Incheon, Gwangju, Daejeon, and Ulsan), and the least of the participants were in the capital city (Seoul), accounting for 12.0%.

The general characteristics of each group identified through the group-based trajectory analysis are presented in Table 2. Of the three groups, the “Persistent Low” group had the highest proportion of males and was the youngest group. Moreover, this group had the highest proportion of participants residing in the capital city (Seoul). Alternatively, the “Persistent High” group had a significantly higher proportion of females and the largest number of participants aged between 65 and 75 years. Additionally, this group had the highest proportion of participants residing in rural areas.

**Table 2:** Descriptive statistics of the study participants.

Category	Total	Expenditure group (n, %)			*p*
	n = 2,862				
	(%)	Persistent Low Expenditure group	Expenditure Increasing group	High Expenditure group	
		(*n* = 2,185)	(*n* = 398)	(*n* = 279)	
Gender					
Male	1,217 (42.5)	659,288 (1,077,675)	668,602 (493,686)	3,272,894 (2,415,196)	0.0087
Female	1,645 (57.5)	688,749 (892,205)	821,277 (586,196)	3,513,784 (2,959,482)	
Age group					
45–54	323 (11.3)	524,704 (600,126)	786,749 (592,085)	3,555,990 (3,049,068)	0.002
55–64	702 (24.5)	691,630 (1,188,840)	801,469 (562,149)	3,905,392 (3,087,670)	
65–74	1,108 (38.7)	700,266 (1,019,369)	692,470 (463,984)	3,092,435 (2,161,104)	
75 and up	729 (25.5)	699,424 (827,884)	842,895 (667,697)	3,517,511 (3,264,052)	
Education level					
Elementary school graduate	1,289 (45.0)	703,632 (1,002,611)	770,744 (562,477)	3,446,400 (2,773,109)	<0.0001
Middle school graduate	534 (18.7)	409,893 (658,130)	625,510 (429,566)	2,656,426 (2,858,642)	
High school graduate or above	1,039 (36.3)	692,549 (945,907)	767,831 (563,821)	3,092,063 (2,032,412)	
Type of medical insurance coverage					
National Health Insurance	2,632 (92.0)	649,287 (922,422)	826,381 (596,215)	3,996,976 (3,041,219)	0.0046
Medical Aid	230 (8.0)	669,021 (1,040,894)	723,602 (525,144)	3,545,964 (3,448,346)	
Marital status					
Married	2,034 (71.1)	677,405 (1,031,742)	740,242 (542,059)	3,492,828 (2,889,321)	0.0048
Single, separated, divorced	828 (28.9)	671,538 (839,919)	823,253 (592,511)	3,194,711 (2,325,286)	
Self-rated health status					
Poor	922 (32.2)	814,035 (1,135,166)	949,005 (633,621)	3,548,055 (2,778,454)	<0.0001
Fair	1,224 (42.8)	624,504 (741,135)	689,218 (472,236)	3,504,707 (2,929,507)	
Good	716 (25.0)	607,637 (1,105,870)	620,101 (525,933)	2,839,164 (2,342,329)	
Income level					
Low	875 (30.6)	613,179 (816,733)	793,858 (580,014)	3,010,408 (1,818,806)	0.0023
Under-middle	704 (24.6)	712,219 (942,606)	777,626 (548,302)	3,364,340 (2,508,090)	
Middle	550 (19.2)	749,766 (1,355,236)	730,753 (565,337)	3,430,062 (3,008,393)	
Upper-middle	394 (13.8)	668,759 (930,984)	770,588 (588,855)	3,914,103 (2,984,113)	
High	339 (11.8)	653,235 (710,314)	715,516 (509,657)	3,909,249 (3,913,568)	
Region^†^					
Capital city	329 (12.0)	668,338 (781,019)	780,837 (464,760)	3,200,091 (2,238,371)	<0.0001
Metropolitan city	783 (27.0)	727,122 (1,261,655)	822,538 (616,243)	3,323,308 (2,298,604)	
Rural area	1,750 (61.0)	653,822 (860,963)	737,724 (541,698)	3,503,548 (3,020,194)	

^†^Capital City: Seoul, Metropolitan City: Busan, Daegu, Incheon, Gwangju, Daejeon, Ulsan.

The results of the multinomial logistic regression analysis are presented in Table 3. Compared with the “Expenditure Increasing” group, the “Persistent Low Expenditure” group (ref: Expenditure Increasing group) showed statistically significant associations with certain factors. Individuals in metropolitan cities had 0.493-fold higher odds (<0.05) of using medical expenditures than those residing in the capital region.

**Table 3:** Results of multinomial logistic regression analysis.

Category	Expenditure increasing group			High expenditure group		
	(vs. persistent low expenditure group)			(vs. persistent low expenditure group)		
	Coef.	OR	95% CI	Coef.	OR	95% CI
Gender (ref: male)						
Female	0.28^*^	1.32	1.02–1.69	0.43^**^	1.54	1.15–2.07
Age group (ref: 45–54)						
55–64	0.37	1.45	0.93–2.24	0.88^**^	2.41	1.31–4.43
65–74	0.55^*^	1.73	1.12–2.67	1.20^***^	3.31	1.81–6.03
75 and up	0.46	1.58	0.99-2.53	1.20^***^	3.31	1.75–6.24
Education level (ref: elementary school graduate)						
Middle school graduate	−0.07	0.93	0.69–1.28	0.2	1.12	0.86–1.74
High school graduate or above	−0.21	0.81	0.60–1.10	−0.04	0.96	0.67–1.37
Marital status (ref: married)						
Single, separated, divorced	−0.14	0.87	0.67–1.14	−0.63^***^	0.53	0.38–0.74
Self-rated health status (ref: poor)						
Fair	−0.07	0.94	0.73–1.2	−0.85^***^	0.43	0.31–0.57
Good	−0.46^**^	0.63	0.46–0.86	−1.19^***^	0.31	0.21–0.44
Income level (ref: Low)						
Under-middle	0.29	1.33	0.99–1.78	0.47^*^	1.60	1.12–2.28
Middle	0.11	1.11	0.79–1.57	0.65^**^	1.92	1.3–2.84
Upper-middle	0.01	1.00	0.68–1.50	0.29	1.35	0.84–2.16
High	0.65^**^	1.91	1.28–2.86	1.02^***^	2.78	1.73–4.47
Region^†^ (ref: capital city)						
Metropolitan city	0.53^**^	1.69	1.11–2.59	0.31	1.36	0.84–2.19
Rural area	0.52^**^	1.69	1.13–2.52	0.41	1.51	0.97–2.34

^†^Capital City: Seoul, Metropolitan City: Busan, Daegu, Incheon, Gwangju, Daejeon, Ulsan. *p <0.05, **p <0.01, ***p <0.001.

The region was not significant in the result of comparing the “Expenditure Increasing” group with the “High Expenditure” group (ref: Expenditure Increasing group).

After performing multinomial logistic regression, the researchers conducted a doubly robust IPW analysis to adjust for demographic, socioeconomic, and health-related factors. This analysis aimed to examine the relationship between regions and medical expenditure trajectory groups while accounting for potential confounding variables. The IPW analysis allowed them to estimate the ATE in this context.

The results of the doubly robust IPW analysis showing the ATE are presented in Table 4. The odds ratio (OR) for the likelihood of being in the “Expenditure Increasing” group compared with the “Persistent Low Expenditure” group was higher for individuals residing in the “metropolitan city” than those in the “capital region” (OR = 1.07; 95% CI: 1.03-1.12). Additionally, it was higher for individuals residing in the “rural area” than those in the “capital region” (OR = 1.07; 95% CI: 1.03-1.11).

**Table 4:** Results of doubly robust inverse probability weighting analysis.

		OR	95% CI	*P*-value
Expenditure increasing group (vs. persistent low expenditure group)				
	Capital city	1.00		
	Metropolitan city	1.07	(1.03–1.12)	0.001
	Rural area	1.07	(1.03–1.11)	<0.001
High expenditure group (vs. persistent low expenditure group)				
	Capital city	1.00		
	Metropolitan city	1.03	(0.99–1.08)	0.067
	Rural area	1.05	(1.01–1.08)	0.008

^†^Capital City: Seoul, Metropolitan City: Busan, Daegu, Incheon, Gwangju, Daejeon, Ulsan.

Moreover, the OR for the likelihood of being in the “High Expenditure” group compared with the “Persistent Low Expenditure” group was higher for individuals in the “rural area” than those in the “capital region” (OR = 1.05; 95% CI: 1.01-1.08).

## 4 Discussion

Considering the unique characteristics of each local community in designing effective chronic disease management programs is crucial. Identifying the factors that vary across regions is essential for developing appropriate intervention strategies (27, 28). Hypertension, being a highly prevalent chronic condition, requires timely management and intervention to prevent complications such as chronic heart failure or stroke. Thus, a nationwide approach to its management is necessary (29).

This study is notable for examining the retrospective 5-year trajectory of medical expenditure for patients with hypertension. Through trajectory analysis, medical expenditure was categorized into three groups: “Expenditure Increasing”, “Persistent Low Expenditure”, and “High Expenditure”. The likelihood of being in the “High Expenditure” group (compared with the “Persistent Low Expenditure” group) was 1.05-fold higher for individuals residing in rural areas than those in the capital region (Seoul), whereas the difference for those in metropolitan cities (Busan, Daegu, Incheon, Gwangju, Daejeon, and Ulsan) was not significant. Similarly, the likelihood of being in the “Expenditure Increasing” group (compared with the “Persistent Low Expenditure” group) was 1.07-fold higher for individuals in rural areas than those in the capital region. Additionally, individuals in metropolitan cities were 1.07 times more likely to be in the “Expenditure Increasing” group (compared with the “Persistent Low Expenditure” group) than those in the capital region. Previous studies have also indicated that residents of metropolitan cities in Korea manage hypertension more effectively compared to those in rural areas (30).

Examining Figure 2, which displays the medical expenditure trajectory groups extracted through trajectory analysis, we observe that the “High Expenditure” group consistently incurs high medical expenditures over the 5-year period. This group is characterized not only by individual health status but also by personal preferences, including economic status. These individuals continue to incur high medical expenditures based on their health status; however, they still tend to spend a significant amount on healthcare even when their economic status is favorable (31). Conversely, the “Expenditure Increasing” group initially incurs medical expenditures similar to the “Persistent Low Expenditure” group; however, from the second year onward, a steep upward trajectory in medical expenditures is observed. For this group, a mix of preferences may be involved; however, from another perspective, it can be inferred that their health status has rapidly deteriorated.

Health disparities are influenced by complex factors, including medical disparities and social determinants (14, 15). In this study, we examined regional differences in medical expenditure among patients with hypertension. Regions can directly or indirectly influence predisposing (age and gender), enabling (economic status), and needs factors (health status) that affect medical utilization. These factors are also related to healthcare utilization and, consequently, medical expenditure. Therefore, investigating regional disparities in medical expenditure among patients with hypertension is essential.

This study highlights regional disparities in medical expenditure, indicating variations in factors such as medical infrastructure, income levels, and health status, which influence healthcare utilization. These differences in healthcare utilization may indicate the presence of health disparities among regions.

Health inequalities are more influenced by social structures, including income levels, educational attainment, employment status, and gender, rather than solely by biological or genetic factors (32). Social structures encompass the formations resulting from socioeconomic status, including factors such as health behavior environments, material environments, and psychosocial environments, all influenced by these factors. Regions encapsulate these elements and, particularly in South Korea, where urban areas have experienced rapid and radical development and growth, stark regional disparities are evident.

Park (32) confirmed the existence of regional health inequalities not only between the capital region and metropolitan areas but also within urban areas at different levels. The study highlighted the need for policies to address these disparities. Another study investigating rural–urban health disparities reported that regions can encompass various health-related factors, including poverty, racial issues, and structural inequalities (33). A study investigating the correlation between the location of infectious disease testing sites and health disparities mentioned that geographic accessibility contributes to health disparities (34). A study focusing on health utilization variations between urban and rural areas showed that residents in urban areas have better opportunities for outpatient and inpatient care, indicating that disparities based on residential location persist (35). Furthermore, other studies exploring health inequalities between rural and urban areas stated the existence of disparities and emphasized how regions reflect structural, economic, and social differences within society (36). Moreover, previous studies on chronic disease-related regional health disparities reported that areas with higher urbanization levels have better hypertension management and lower prevalence rates (37). Other studies have investigated chronic disease-related regional disparities (38). These studies collectively emphasize that regions are critical variables that influence the factors of predisposing, enabling, and need related to healthcare utilization and health outcomes.

The Korean government aimed to extend healthy life expectancy and enhance health equity by strengthening health promotion projects for vulnerable families and improving health disparity monitoring. In 2018, the Ministry of Health and Welfare launched a plan to address regional healthcare disparities and enhance coverage. The plan emphasizes the role of primary care physicians in mitigating healthcare quality disparities by considering patients’ circumstances. However, continuous management of chronic diseases through medical institutions remains insufficient (39).

One of the opinion to reduce regional disparities is to shift from focusing on averages to addressing disparities. This means developing and implementing detailed strategies to reduce healthcare disparities, establishing robust systems for performance monitoring and reporting, and promoting innovative primary care initiatives (39). Another previous article mentioned that it is important to holistically include socio-economic status, community development, and healthcare infrastructure. It is necessary to consider long-term budgeting to accomplish these policies and long-term planning (40).

A fundamental understanding of spatial attributes is essential to effectively address regional health inequalities. First, examining the availability of local healthcare services and the distribution of medical facilities within the capital region and metropolitan areas is significant. Additionally, one must be aware that regional social structures encompass health behavior, physical, and psychological environments formed according to socioeconomic status and class. In other words, the structure of the local community, where we live, is composed of various factors that influence health. Therefore, the role of local governments is just as crucial as that of the central government. Starting with the improvement of factors causing inequalities within the local community, efforts should be made to gradually reduce regional disparities.

This study had several limitations. First, it did not adequately reflect health status, particularly the duration of hypertension, which could have influenced the medical expenditure trajectories. However, the use of doubly robust estimation helped to partially compensate for this limitation. Furthermore, when examining the core observation groups including the “Expenditure Increasing” and “Persistent Low Expenditure” groups, it was observed that their initial medical expenditure levels were similar. Therefore, this aspect may have been somewhat compensated for. Second, this study did not capture the changes in individual characteristics and the severity of hypertension over time. However, trajectory analysis reflected such overall changes to some extent; therefore, it may have addressed this limitation to an appropriate degree.

In conclusion, this study has shed light on the regional disparities in medical expenditure among patients with hypertension. Particularly, when comparing the “Expenditure Increasing” group with the “Persistent Low Expenditure” group, it was observed that individuals residing in rural areas spent more on medical expenditures than those residing in the capital region. This suggests that the rural group has been receiving relatively inadequate management for hypertension compared with the capital region group. These regional disparities can be interpreted as indicators of health inequality; to address this, efforts should be made through relevant policies for reducing the imbalance in social structures and resource disparities between regions.

## Data availability statement

Publicly available datasets were analyzed in this study. This data can be found at: https://www.khp.re.kr:444/.

## Ethics statement

The studies involving human participants were reviewed and exempt by the Institutional Review Board of Yonsei University (1041849-202211-SB-214-01). The studies were conducted in accordance with the local legislation and institutional requirements. The ethics committee/institutional review board waived the requirement of written informed consent for participation from the participants or the participants’ legal guardians/next of kin because the data is secondary, publicly available.

## Author contributions

G-HK: Data curation, Formal analysis, Methodology, Writing – original draft. J-SS: Project administration, Writing – review & editing. J-WN: Data curation, Methodology, Project administration, Writing – review & editing. W-RL: Methodology, Writing – review & editing. K-BY: Conceptualization, Funding acquisition, Methodology, Supervision, Writing – review & editing.
